# Evaluating Basigin as a Potential Biomarker of Blood–Brain Barrier Dysfunction in Cerebral Amyloid Angiopathy

**DOI:** 10.1111/nan.70064

**Published:** 2026-02-18

**Authors:** Arno Stellingwerf, Davita Bosveld, Lieke Jäkel, Anna M. De Kort, Benno Küsters, Catharina J. M. Klijn, Floris H. B. M. Schreuder, H. Bea Kuiperij, Marcel M. Verbeek

**Affiliations:** ^1^ Department of Neurology, Donders Institute for Brain, Cognition and Behaviour Radboud University Medical Center Nijmegen the Netherlands; ^2^ Department of Pathology Radboud University Medical Center Nijmegen the Netherlands; ^3^ Department of Human Genetics Radboud University Medical Center Nijmegen the Netherlands

**Keywords:** basigin, biomarkers, blood–brain barrier, cerebral amyloid angiopathy, cerebrospinal fluid, choroid plexus

## Abstract

**Aims:**

Blood–brain barrier (BBB) dysfunction may be involved in the pathophysiology of neurodegenerative disorders, including sporadic cerebral amyloid angiopathy (CAA). Because basigin (BSG) may induce activity of matrix metalloproteinases and thereby BBB breakdown, we investigated BSG expression in CAA brain tissue with immunohistochemistry and as cerebrospinal fluid biomarker for BBB dysfunction in patients with CAA.

**Methods:**

Using immunohistochemistry, we quantified BSG expression within the cortical microvasculature of the temporal lobe of 50 CAA patients (16 with intracerebral haemorrhage [CAA‐ICH] and 34 without intracerebral haemorrhage [CAA‐NH]) and 35 controls. To investigate whether BSG is expressed at another brain barrier, choroid plexus tissue was qualitatively assessed. Additionally, we compared cerebrospinal fluid levels of BSG between 40 CAA patients and 27 healthy controls using ELISA.

**Results:**

Cortical vessels, in particular capillaries, were positive for BSG. Median %area of BSG expression was increased in CAA cases (1.02%, IQR [0.63–1.68]) compared with controls (0.65%, IQR [0.43–1.01], *p* = 0.013). Moreover, we observed more BSG staining in CAA‐NH (1.23%, IQR [0.90–2.0]) than in CAA‐ICH (0.58%, IQR [0.24–1.00], *p* = 0.001) and controls (*p* < 0.001). Choroid plexus epithelial cells showed apical BSG expression, whereas endothelial cells were negative. The median BSG concentration in CSF was decreased in CAA (11.0 ng/mL, IQR [10.1–13.8]) compared with controls (13.00 ng/mL, IQR [11.5–14.5], *p* = 0.02).

**Conclusions:**

CSF concentrations of BSG may be related to altered expression at the BBB; the BSG concentrations in CSF may thus serve as a biomarker of BBB function, although a contribution of choroid plexus epithelial BSG cannot be entirely excluded. Independent cohorts are needed to replicate these observations before we can conclude that reduced CSF concentrations of BSG may indicate an alteration of the BBB in patients with CAA.

## Summary

- Cerebrovascular BSG staining is increased in CAA‐NH cases compared to CAA‐ICH and control.
- The CSF BSG concentration is decreased in patients with CAA compared to control.
- Altered cerebrovascular BSG regulation may contribute to BBB‐dysfunction in CAA.

## Graphical abstract

Basigin (BSG) is a potential biomarker for blood‐brainbarrier dysfunction since link via matrix metalloproteinases. In a clinical cohort and in post‐mortem cortical tissue, we compared the CSF levels and vascular BSG immunoreactivity between patients with Cerebral amyloid angiopathy (CAA) and control cases. The CSF BSG concentration is decreased in patients with CAA compared to control while vascular BSG is increased in CAA cases. Because the choroid plexus also expresses BSG the specificity of this BBB‐biomarker is reduced.

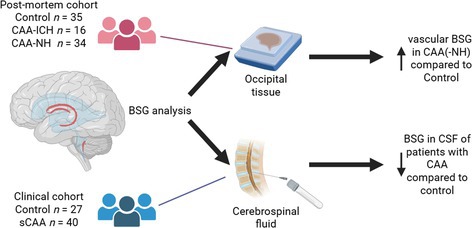

**Abbreviations**
Aβ: amyloid‐Beta
ad
: Alzheimer's disease
BBB: blood–brain barrier
BBI: Borge Bunge Institute
BCSFB: blood–CSF barrier
BSG: basigin
CAA: sporadic cerebral amyloid angiopathy
CAA‐ICH: CAA patients with intracerebral haemorrhage(s)
CAA‐NH: CAA nonhaemorrhagic
CapCAA: capillary CAA
CMB: cerebral microbleed
CNS: central nervous system
CSF: cerebrospinal fluid
DCE‐MRI: dynamic contrast‐enhanced MRI
ECM: extracellular matrix
EMMPRIN: extracellular matrix metalloproteinase inducer
FFPE: formalin‐fixed, paraffin‐embedded
IQR: interquartile range
MMP: matrix metalloproteinase
NBB: Netherlands Brain Bank
NVU: neurovascular unit
p‐tau: phosphorylated tau
Qalb: CSF/serum albumin quotient
RD: reagent diluent
RUMC: Radboud University Medical Center Nijmegen
SD: standard deviation
SVD: small vessel disease
t‐tau: total tau
UMCU: University Medical Center Utrecht
%areaBSG: percentage area stained for BSG

## 1 Introduction

Sporadic cerebral amyloid angiopathy (CAA) is a prevalent disease in which amyloid‐beta (Aβ) peptides accumulate in the leptomeningeal and cortical arterioles, capillaries and venules [1, 2]. Patients with CAA have an increased risk of lobar intracerebral haemorrhages (ICH) and developing cognitive impairment and may also experience transient focal neurological episodes [3]. The Aβ peptides in CAA are hypothesised to result in vascular damage [4, 5, 6, 7, 8]. Indeed, MRI and histology studies have revealed vascular deficiencies in CAA, altered extracellular matrix (ECM) composition, decreased clearance of waste products and endothelial cell tight junction alterations potentially resulting in increased blood–brain barrier (BBB) permeability [4, 5, 6, 7, 8].

The BBB is a metabolic and physical semipermeable barrier between the peripheral blood circulation and the central nervous system (CNS) [9]. Endothelial cells, pericytes and astrocytic end‐feet contribute to the BBB and communicate with adjacent microglia and neurons to form the neurovascular unit (NVU) [9].

Several methods to assess BBB‐(dys)function are currently used. BBB‐dysfunction can be assessed with immunohistochemistry (IHC) on postmortem tissue, demonstrating extravasated (serum) proteins such as fibrinogen, thrombin and albumin, along with aberrant vessel morphology and cell–cell connections [4, 10, 11]. In vivo assessment of BBB leakage can be evaluated with dynamic contrast‐enhanced MRI (DCE‐MRI), but subtle leakages are challenging to detect [12]. The most frequently used fluid biomarker for BBB‐dysfunction is the ratio of the albumin concentration in cerebrospinal fluid (CSF) to serum (Qalb). However, because CSF is predominantly produced in the choroid plexus, the Qalb either reflects (dys)function of the blood–CSF barrier (BCSFB) located in the choroid plexus or aberrant CSF flow. Therefore, the Qalb has erroneously been used as a biomarker of BBB (dys)function [13, 14, 15, 16, 17]. To define BBB‐specific molecular biomarkers, proteins should be specifically and mechanistically linked to the BBB and not to the BCSFB. However, cells constituting the BCSFB and BBB express many similar proteins [18, 19, 20, 21]. Accordingly, when assessing a fluid biomarker for BBB‐dysfunction in CSF or blood, other sources of proteins, for example, the parenchyma or BCSFB, can contribute to the measured concentration, potentially introducing information bias. Therefore, biomarker sources should be evaluated, and a mechanistic link of the biomarker to the BBB should be established.

BBB impairment has been linked to aberrant matrix metalloproteinase (MMP) regulation in various neurological disorders, where the ability of MMPs to degrade tight junction and extracellular matrix proteins may destabilise the BBB [22, 23, 24, 25]. Several studies have demonstrated MMP‐induced loss of claudin‐5, occludin and zonula occludens‐1 proteins in rats in the context of ischaemia and traumatic brain injury [26, 27, 28]. Aberrant expression of MMPs and their inhibitors has been associated with CAA [29, 30, 31, 32].

In this study, we aim to evaluate the protein basigin (BSG), an inducer of MMP function, as a potential BBB‐dysfunction biomarker in CAA. This glycoprotein of the immunoglobulin superfamily, also known as CD147 and as extracellular matrix metalloproteinase inducer (EMMPRIN), is involved in both ECM remodelling and Aβ metabolism via activating MMPs [33, 34, 35, 36]. We hypothesise that increased cerebrovascular BSG levels may play a role in the destabilisation of the BBB via activation of MMP proteins and are reflected by increased expression in the cerebral vasculature and the CSF of CAA patients. In the human CNS, BSG expression is observed in brain endothelial cells [25, 37]. BSG has both a transmembrane and soluble form and can be found in serum and CSF [38]. Because of its specific expression in cerebrovascular endothelial cells, its mechanistic relevance and its presence in a soluble form in body fluids, BSG has previously been proposed as a potential BBB biomarker. However, BSG expression at the human BCSFB has not been evaluated before [39, 40]. Therefore, it is unknown whether the BCSFB could influence the CSF concentrations of BSG.

To assess the role of BSG in CAA and its potential as a BBB (dys)function marker, we studied the expression of BSG in cerebral temporal brain tissue of both CAA and control cases and in choroid plexus tissue. Furthermore, we quantified CSF levels of BSG in patients with CAA and healthy controls.

## 2 Materials and Methods

### 2.1 Immunohistochemistry Cohort

Human postmortem formalin‐fixed, paraffin‐embedded (FFPE) temporal lobe tissue was obtained from the Radboud University Medical Center Nijmegen (RUMC), the Netherlands Brain Bank (NBB), the Borge Bunge Institute (BBI) and the University Medical Center Utrecht (UMCU). CAA cases with intracerebral haemorrhage (CAA‐ICH) and nonhaemorrhagic CAA (CAA‐NH) cases were selected based on neuropathological assessments in autopsy reports and the presence of moderate‐to‐severe CAA [31]. In addition, information on the presence of capillary CAA (CapCAA) and *APOE ɛ4* allele carriership was retrieved from the autopsy reports. In those cases without information on the *APOE* genotype, immunohistochemistry‐based phenotyping data were used (Jäkel et al., in preparation). In short, these cases were stained using an apoE *ɛ*4 antibody (Abcam, 811601, 1:150 dilution), and when the staining was negative for apoE *ɛ*4, staining using a pan‐apoE antibody (Abcam, AB1907, dilution 1:500) was performed as a control to check if apoE *ɛ*4 was present in the tissue section. Information on the presence of capCAA and *APOE ɛ4* allele carriership (based on either genotyping or phenotyping data) is reported in Table 1. Age‐ and sex‐matched control cases were selected based on the absence of neurological disorders or amyloid pathologies in autopsy reports (Table 1). Two independent raters were blinded to the diagnosis and determined the per case CAA burden according to the Olichney scoring system [41]. A third reader was consulted if there was no consensus.

**TABLE 1:** Demographics and CAA grade of temporal lobe immunohistochemistry cohort.

	Controls *N* = 35	CAA (all) *N* = 50	CAA‐ICH^a^ *N* = 16	CAA‐NH *N* = 34	*p* *
Age (years; mean ± SD)	78.7 (8.2)	78.0 (9.5)	76.8 (7.0)	78.5 (10.3)	0.713^b^
Sex (m/f)	18/17	23/27	8/8	15/19	0.847^c^
Biobank source	IBB: *n* = 7, NBB: *n* = 14, RUMC: n = 14	IBB: *n* = 12, NBB: *n* = 18, RUMC: *n* = 17, UMCU: *n* = 3	IBB: *n* = 6, NBB: *n* = 4, RUMC: *n* = 3, UMCU: *n* = 3	IBB: *n* = 6, NBB: *n* = 14, RUMC: *n* = 14	
*APOE ɛ4* carriership^e^ (no/yes/N.D.)	16/4/15	9/17/24	5/1/10	4/16/14	**< 0.001** ^d^
capCAA reported (no/yes/N.D.)	0/3/32	26/18/6	7/4/5	19/14/1	0.129^d^
CAA grade (median, IQR)	0 [0–0]	3 [2]	4 [2–4]	3 [2–4]	**< 0.001** ^c^

*Note:* Bold values represent significant *p*‐values.

Abbreviations: *APOE ɛ4*, apolipoprotein E ɛ4; capCAA, capillary cerebral amyloid angiopathy; CAA‐ICH, cerebral amyloid angiopathy with intracerebral haemorrhage; CAA‐NH, cerebral amyloid angiopathy non haemorrhagic; IBB, Institute Born‐Bunge; IQR, interquartile range; NBB, Netherlands Brain Bank; RUMC, RadboudUMC; SD, standard deviation; UMCU, University Medical Center Utrecht.

^a^ Temporal tissue of two CAA‐ICH cases was unavailable; therefore, frontal lobe tissue was used in these cases.

^b^ Unpaired *t* test.

^c^ Fisher's exact test.

^d^ Chi‐square test.

^e^ Based on APOE genotyping or phenotyping analysis.

* *p* value for the comparison between control, CAA‐ICH and CAA‐NH.

To qualitatively assess if BSG is expressed by the BCSFB, we were kindly provided with three random surplus choroid plexus tissues from the RUMC Department of Pathology. These tissues were randomly selected; therefore, disease, age and sex were unknown.

### 2.2 Immunohistochemical BSG Staining and Image Analysis

After 4‐μm‐thick FFPE temporal lobe sections were mounted on object glasses (VWR international BV, Amsterdam, the Netherlands, #631‐0108), sections were deparaffinised and hydrated by submerging once in xylene, twice in 100% ethanol and once in 70% ethanol. Endogenous peroxidase activity was blocked by incubation for 15 min with 3% H_2_O_2_ in methanol at room temperature. Next, antigen retrieval was performed by boiling slides for 10 min in EDTA buffer (pH 8.5). Incubation steps were always followed by three washes with phosphate‐buffered saline, pH 7.4. Sections were blocked for 2 h with 5% normal swine serum in PBS and subsequently incubated overnight with polyclonal rabbit anti‐BSG antibodies diluted 1:400 (Proteintech Group Inc., Rosemont, Illinois, USA; #11989‐1‐AP) at 4°C. Next, sections were incubated with biotinylated polyclonal goat anti‐rabbit diluted 1:400 (Vector Laboratories, Newark, California, USA, #BA‐1000) for 30 min at RT. Sections were incubated with preincubated AB‐complex (100:1:1 miliQ: component A: component B; Vector Laboratories, #PK‐6100) for 30 min. Hereafter, sections were incubated with 3,3′‐diaminobenzidine (DAB; Vector Laboratories, #34002) for 7 min. Excess DAB was removed, and sections were counterstained with the nuclear staining haematoxylin (Sigma‐Aldrich, #MHS16‐500ML). Dehydration was performed in reverse order of the hydration procedure, and sections were mounted with Pertex (VWR #VWRKAM‐0801) and dried overnight. The primary antibody was omitted in the negative control.

Stained tissue sections were imaged with a 3DHistech P100 scanner (3DHistech, Budapest, Hungary). Ten images of the grey matter were made at 20× magnification in a zig‐zag pattern across the deepest sulci and analysed. The images were automatically processed with FIJI ImageJ (Wayne Rasband, NIH, USA, Version 1.8.0_202), first by using colour deconvolution, separating the haematoxylin and the DAB staining. A Sauvola local threshold [42], with a radius of 20 (pixel units) and *k* value of 0.5, was used to filter out low background staining. Subsequently, the stained tissue area was calculated per section (in %). Two internal controls were applied in each batch to correct for possible batch variability.

### 2.3 CSF Cohort Characteristics

We included patients with probable CAA, diagnosed according to the modified Boston 1.5 criteria [43] and age‐ and sex‐matched controls from the BIONIC (BIOmarkers for cogNitive Impairment due to Cerebral amyloid angiopathy; www.radboudumc.nl/BCS) study and CAFE (Cerebral Amyloid angiopathy Fluid biomarkers Evaluation) study [44]. The local ethical committee approved the study protocols (file numbers 2017‐3810 and 2017‐3605), and all participants provided written informed consent. Participants underwent lumbar puncture to collect CSF and venipuncture to retrieve blood samples. CSF was collected in polypropylene tubes, centrifuged for 10 min at 800 × g, aliquoted and stored at −80°C. Additionally, from previous publications, information on CSF and serum levels for albumin (to determine Qalb; performed using nephelometry with an Atellica Neph 630 analyser) and CSF levels of Aβ40, Aβ42, total tau (t‐tau), phosphorylated tau (p‐tau) (all four determined using lumipulse chemiluminescent immune assays, Fujirebio, Ghent, Belgium), MMP‐2 (ELISA, R&D systems, Minneapolis, Minnesota, USA, #DY902, with 6× diluted samples), MMP‐9 (ELISA, R&D systems, 896098, 2× diluted samples) and MMP‐14 (ELISA, Finetest, Wuhan, China, EH0369, 4× diluted samples) were available [30, 45, 46]. Because ad and CAA pathologies frequently co‐occur, we estimated the prevalence of ad pathology in our CAA‐cohort, based on ATN profiling [44, 47]. We categorised the CAA group into an ATN positive (ATN+) and ATN negative (ATN−) group using our local cut‐off values for CSF Aβ42 (A+, < 659 pg/mL), CSF p‐tau (T+, > 64 pg/mL) and CSF t‐tau (N+, > 400 pg/mL). Participants underwent a standardised 3‐T brain MRI to determine the lobar cerebral microbleed (CMB) count and small‐vessel disease (SVD) burden score, as previously described [45]. An overview of patient characteristics is shown in Table 2.

**TABLE 2:** Demographics and clinical characteristics of CSF donors with sporadic CAA and controls.

	Controls (*n* = 27)	CAA patients (*n* = 40)	*p*
Age (years; mean ± SD)	71.4 ± 6.8	71.7 ± 6.0	0.848^b^
Sex (m/f)	14/13	21/19	0.958^c^
History of CAA‐ICH (%)	N.A.	9 (22.5%)	N.A.
CMB count (median, [IQR])	0 [0–0]	9 [4–31]	**< 0.001** ^e^
SVD burden score (median, [IQR])	1.0 [1–1]	4.0 [3.2–5]	**< 0.001** ^d^
Qalb (mean ± SD)^a^	6.53 ± 1.65	6.65 ± 2.08	0.80^b^
CSF Aβ40 (ng/mL, mean ± SD)	13.2 ± 3.8	8.3 ± 2.3	**< 0.001** ^b^
CSF Aβ42 (pg/mL, median [IQR])	1069 [805–1326]	382 [288–486]	**< 0.001** ^b^
CSF t‐tau (pg/mL, median, [IQR])	362 [281–490]	421 [281–536]	0.23^e^
CSF p‐Tau (pg/mL, median, [IQR])	41.0 [33.7–63.0]	53.8 [34.6–70.2]	0.186^e^
ATN‐profile (CAA + AD+/CAA + ad−)	N.A.	14/26	N.A.
CSF MMP2 (ng/mL, median, [IQR])^f^	38.6 [31.1–50.6]	29.6 [26.4–32.8]	0.06^e^
CSF MMP9 (ng/mL, median, [IQR])^f^	0.50 [0.37–1.17]	1.10 [0.74–2.34]	0.13^e^
CSF MMP14 (ng/mL, mean ± SD)^f^	3.18 ± 1.02	2.74 ± 0.66	0.32^b^

*Note:* Bold values represent significant *p*‐values.

Abbreviations: Aβ, amyloid beta; CAA, cerebral amyloid angiopathy; CMB, cerebral microbleed; CSF, cerebrospinal fluid; ICH, intracerebral haemorrhage; IQR, interquartile range; MMP, matrix metalloproteinase; P‐tau, phosphorylated tau; Qalb, CSF/serum albumin ratio; SD, standard deviation; SVD, small vessel disease; t‐tau, total tau.

^a^ Available for controls *n* = 27, CAA *n* = 34.

^b^ Unpaired *t* test.

^c^ Chi‐square test.

^d^ Fisher's exact test.

^e^ Mann–Whitney *U* test.

^f^ Data were only available for a selection of the participants [30]. Controls; *n* = 5, CAA patients; *n* = 11.

### 2.4 BSG ELISA

BSG levels in CSF were measured using the Human EMMPRIN/CD147 DuoSet ELISA kit (R&D Systems, Minneapolis, Minnesota, USA, #DY972); 96‐well plates were coated with mouse antihuman BSG antibody (2.0 μg/mL) overnight at RT. Next, plates were washed three times with 0.05% Tween‐20 (polysorbate 20 MPbio, VWR international BV) in PBS. Washing was done after each incubation step. Plates were blocked with reagent diluent (RD; 1% bovine serum albumin (Sigma‐Aldrich, A7030) in PBS) for 1 h at RT. The recombinant BSG standard (31.25–2000 pg/mL), blank (RD) and CSF samples (diluted 1:32) were added to the plates in duplicates and incubated for 2 h at room temperature. Standards and CSF samples were diluted in RD. Next, biotinylated goat antihuman BSG detection antibody (50.0 ng/mL, diluted in RD) was added to each well and incubated for 2 h at RT. Plates were then incubated with streptavidin‐labelled horseradish peroxidase (diluted 1:200) for 20 min at room temperature, after which 3,3′,5,5′‐tetramethylbenzidine substrate solution was added and incubated 20 min at room temperature. The reaction was stopped with 1‐N sulfuric acid. Absorbance was read at 450 nm using a Tecan Infinite F50 ELISA reader. Five CSF pool samples were used as quality controls and were measured in duplicate on each plate to correct for possible variations between plates.

### 2.5 BSG ELISA Validation

The limit of detection (LOD) and lower limit of quantification (LLOQ) of the assay were determined with 15 blanks (RD). To determine intraplate variation, four CSF samples of high, medium and low BSG concentrations (established during kit optimisation) were measured in quadruplicate. The same four CSF samples were used to determine the interassay variation by measuring these in duplicate on three different plates on three different days [48]. Dilutional linearity was determined with four CSF samples of high, medium and low BSG concentrations. Samples were serially diluted from 8‐ to 256‐fold and measured in duplicate. Analysis and calculation of the validation parameters was performed as previously described [48].

### 2.6 Statistical Analysis

Data analysis was done using IBM SPSS Statistics version 29.0 (IBM Corp, Armonk, New York, USA) and Graphpad Prism version 10 (Graphpad Software Inc, San Diego California, USA). For the analysis of the BSG concentration in CSF and of immunohistochemistry data, outliers were identified using Grubbs' test for outliers (*p* < 0.01) and excluded from further analyses. For all parameters collected for the study cohorts, normality of the data was assessed using the Shapiro–Wilk test. If parameters had a Gaussian distribution, they were represented as mean ± SD and groups were compared using parametric tests, for example, an unpaired *t* test. If parameters did not have a Gaussian distribution, they were depicted as medians with interquartile ranges (IQR) and a Mann–Whitney *U* test was used. The distribution of sex and *APOE‐ε4* carriership was compared between groups using the chi‐square test, whereas the distribution of CAA SVD burden score and the temporal CAA grade were compared with Fisher's exact test.

The IHC results were compared as percentage area stained for BSG (%areaBSG). Because differences between the study groups were observed with a Mann–Whitney *U* and Kruskal–Wallis test, we tested for confounding effects with generalised linear models. In this model, we tested for interactions with age, sex and the tissue sources (BBI, NBB and RUMC). Due to the small number of UMCU cases, we combined this source with the RUMC cases in this analysis. This analysis was performed again with the UMCU allocated in the NBB group, and this did not alter the outcome significantly. Because the %areaBSG was right‐skewed, we used the probable Gamma distribution and a Log Link function in the GLM analysis. We used Spearman's test to analyse correlations of %areaBSG with age and with the temporal CAA score. In the CAA cases, the %areaBSG was compared between Capillary CAA positive and negative cases using a Mann–Whitney *U* test. The same test was used to compare the %areaBSG between *APOE ɛ4* carriers and noncarriers in all cases combined, as well as in the individual groups.

The group comparisons of BSG CSF concentrations were corrected for age and sex using multiple linear regression analysis comparing the CAA and control populations. When calculating correlations between BSG concentrations and SVD score, CMB count, Qalb and the CSF levels of Aβ40, Aβ42, t‐ and p‐tau, corrections were applied for the influences of age and sex by performing partial rank correlations with age and sex as independent variables. For comparison of the CSF BSG levels between the CAA + ATN+, CAA + ATN− and control population, a generalised linear model was used with age and sex as covariates. The CAA patient group was divided into three categories based on the median CMB count: *n* = 0, *n* = 1–9, and *n* > 9, and between these groups, the CSF BSG levels were compared using a Kruskal–Wallis test. *p* values < 0.05 were considered statistically significant.

## 3 Results

### 3.1 BSG Expression in the Temporal Cortex

We included 50 patients with CAA, of whom 16 had a prior intracerebral haemorrhage (CAA‐ICH), and 34 had nonhaemorrhagic CAA (CAA‐NH). Additionally, we recruited tissue samples from 35 age‐ and sex‐matched control participants. Characteristics are summarised in Table 2.

**FIGURE 1:**
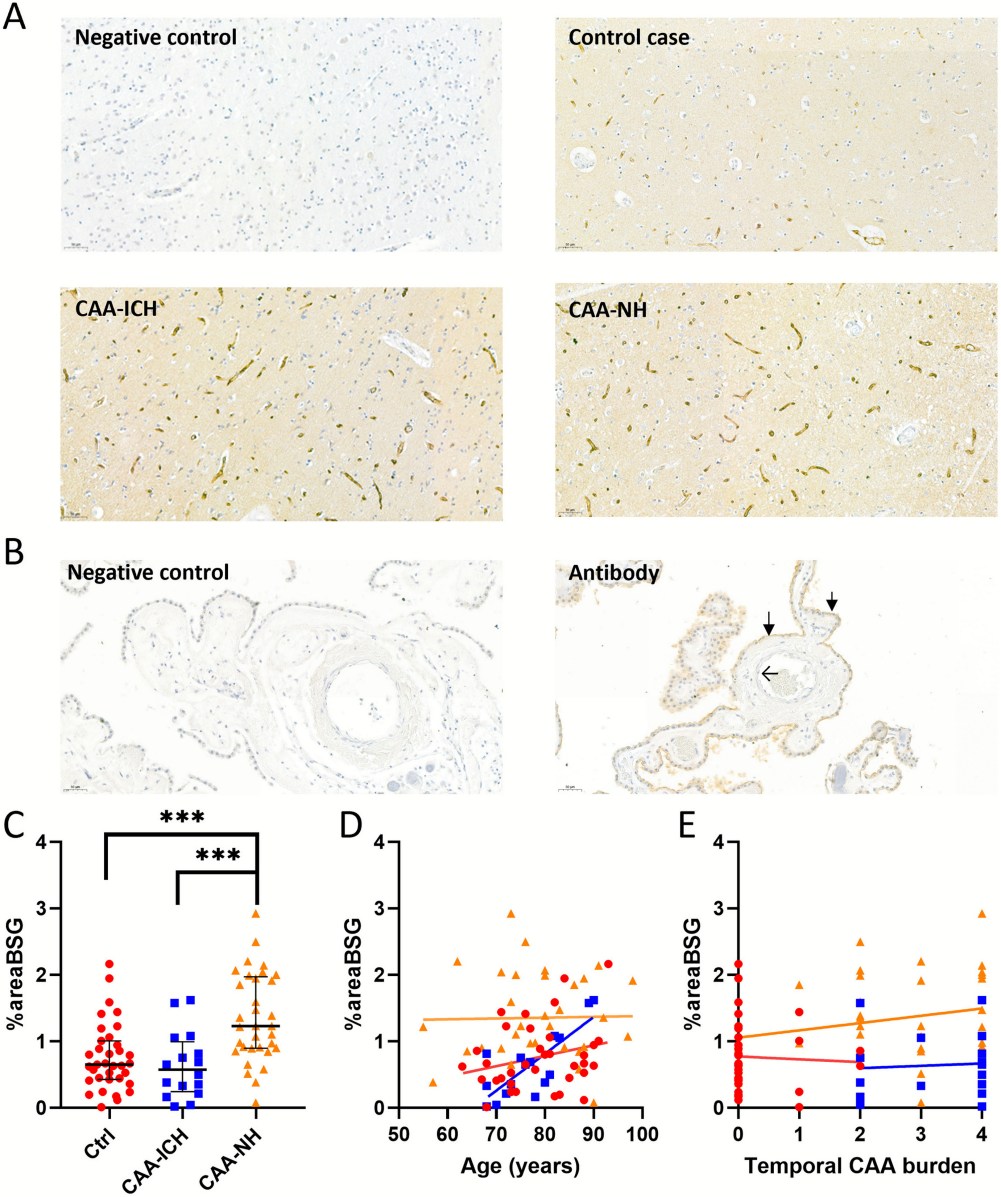
IHC staining of BSG in temporal brain tissue and choroid plexus tissue with subsequent analysis of positive area for BSG (%areaBSG). (A) From left‐to‐right‐top‐to‐down: Representative images of a negative control, which showed no staining, a control case with some cortical staining, a CAA‐ICH case, and a CAA‐NH case, all showing staining in the vasculature, predominantly in capillaries. (B) Negative control of choroid plexus tissue showing no staining for BSG, as compared with BSG staining of epithelial cells of the choroid plexus (closed arrows). The vasculature remains negative in this staining (open arrow). (C) Percentage area stained for BSG (%AreaBSG) was higher in CAA‐NH (orange triangles) than in controls (red circles) and CAA‐ICH (blue squares). (D) The %AreaBSG correlated with age only in the CAA‐ICH group (*p* = 0.041), but not in CAA‐NH or controls. (E) The Olichney CAA severity score (Temporal CAA burden) did not correlate with %AreaBSG. Abbreviations: Ctrl, control group; CAA, cerebral amyloid angiopathy; ICH, intracerebral haemorrhage; NH, nonhaemorrhagic; %areaBSG, percentage positive area for basigin; BSG, basigin. ***p ≤ 0.001.

Overall, BSG immunoreactivity was observed in cortical capillaries of all temporal tissues, with varying degrees of intensity. BSG immunoreactivity in the leptomeningeal and penetrating arterioles and veins was occasionally observed in both control and CAA tissue. We found no parenchymal staining or plaque‐like formation of BSG (Figure 1A). In the choroid plexus, the epithelial cells of the BCSFB stained positively for BSG. The apical side of the epithelial cells showed more immunoreactivity than the basolateral side. We found no BSG in the endothelial cells of the choroid plexus (Figure 1B).

Image analysis showed that %areaBSG was higher in CAA (median 1.02%, IQR [0.63–1.68]) compared with healthy controls (median 0.65%, IQR [0.24–1.01]) (*p* = 0.010). After testing for outliers, one case was removed from the CAA(‐NH) group (4.18%), but this did not influence the results (*p* = 0.013). This outlier was excluded from further analyses. The %areaBSG was higher in the CAA‐NH group (median 1.23%, IQR [0.90–2.0]) in comparison to both the control (*p* < 0.001) and the CAA‐ICH group (median 0.58%, IQR [0.24–1.00], *p* = 0.001, Figure 1C). Adjusting for the covariates age, sex and tissue source did not significantly affect the observed differences between the groups.

In the CAA‐ICH group, we found a significant correlation between age and %areaBSG (*ρ*_sp_ = 0.681, *p* = 0.004), but not in controls (*ρ*_sp_ = 0.227, *p* = 0.191) or the CAA‐NH group (*ρ*_sp_ = −0.006, *p* = 0.975, Figure 1D). We did not find a difference in %areaBSG between *APOE ɛ4* carriers and noncarriers in all groups combined (*p* = 0.626). However, in the CAA‐NH group, the *APOE ɛ4* carriers had significantly less immunoreactivity (1.16%, IQR [0.64]) for BSG than the noncarriers (2.08%, IQR [0.87], *p* = 0.016), whereas the *APOE ɛ4* carriership did not alter the BSG staining in the control population (*p* = 0.179). No association of *APOE ɛ4* carriership and %areaBSG could be calculated in the CAA‐ICH group because there was only a single case *APOE ɛ4 positive*.

We observed no significant correlation between CAA burden and %areaBSG in the individual CAA groups (CAA *ρ*_sp_ = −0.001, 0.992; CAA‐ICH *ρ*_sp_ = 0.268, *p* = 0.335; CAA‐NH *ρ*_sp_ = 0.141, *p* = 0.441; see Figure 1E.) Neither did we find a difference for %areaBSG between CAA cases with CapCAA (median 1.08% IQR [0.84–1.78]) or without CapCAA (median 0.94%, IQR [0.52–1.87], *p* = 0.398).

### 3.2 CSF Concentrations of BSG

The inter‐assay and intra‐assay variation of the BSG ELISA were 5.9% and 1.2%, respectively. The dilutional linearity ranged between 95% and 120% for dilution from 8‐ to 128‐fold. The LOD and LLOQ of the BSG ELISA were respectively 13.4 and 44.7 pg/mL. All CSF samples had a BSG concentration above the LLOQ and a %CV < 20%.

**FIGURE 2:**
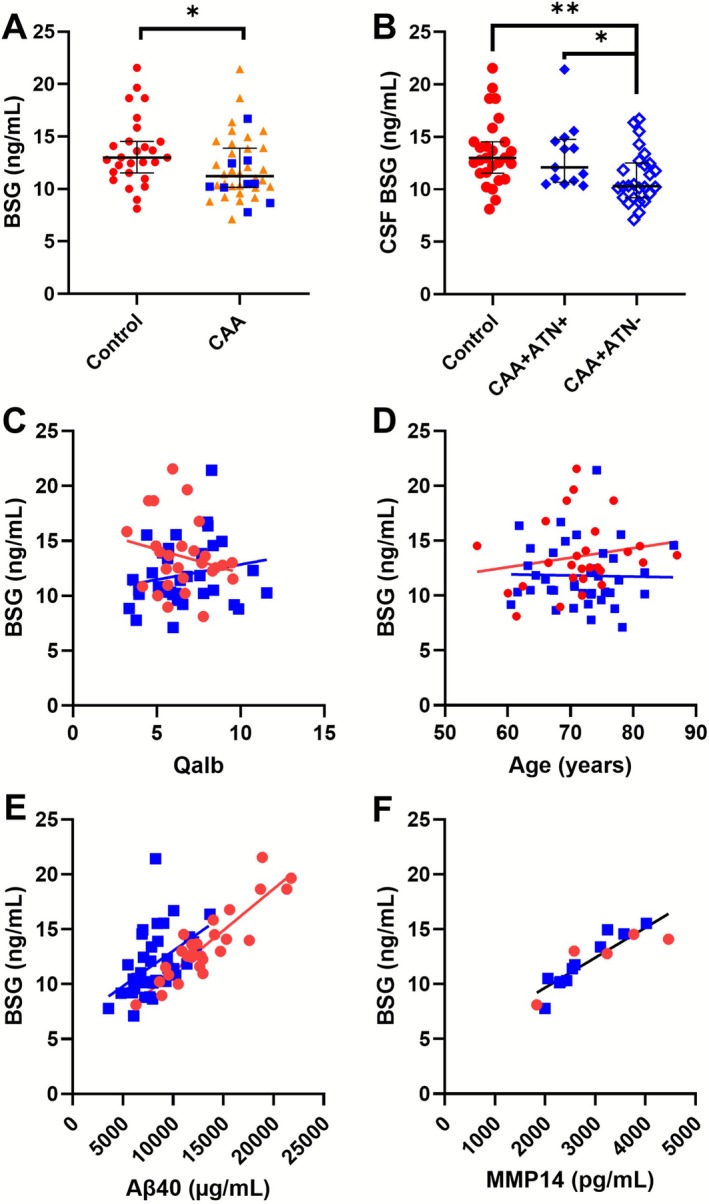
CSF BSG concentrations in controls (red circles) and CAA (both CAA‐NH = orange triangles; CAA‐ICH = blue squares), as well as different correlations depicted in scatterplots. (A) The CSF BSG concentration was lower in CAA (blue squares + orange triangles)) than in controls (red circles) (*p* = 0.02). No difference between CAA‐NH (blue squares) and CAA‐ICH (orange triangles) was detected. (B) CAA + ATN− cases (open blue diamonds) had lower BSG CSF concentrations than both control (red circles, *p* = 0.001) and CAA + ATN+ (filled blue diamonds, *p* = 0.037), independent of age and sex. (C) The concentration of CSF BSG neither correlated with the albumin ratio (Qalb) in controls (*p* = 0.32) nor in CAA patients (*p* = 0.20). (D) No correlation between BSG concentration and age was observed in the control (*p* = 0.31) and CAA cases (*p* = 0.64), (E) BSG correlated with Aβ40 in both controls (*p* < 0.001) and CAA (*p* < 0.001). (F) The CSF BSG concentration correlated with MMP14 in an explorative subcohort of both controls and CAA patients combined (*p* < 0.001). Abbreviations: CSF, cerebrospinal fluid; BSG, basigin; Ctrl, control; CAA, cerebral amyloid angiopathy; Qalb, CSF/blood albumin ratio; Aβ40, amyloid beta 40; MMP14, matrix metallopeptidase 14, * *p* < 0.05, ** *p* = 0.01.

In the CAA group, the median CSF BSG concentration was 11.2 ng/mL (IQR [10.21–13.9]), and in this group, there was one outlier with a BSG concentration of 27.3 ng/mL. No outliers were present in the control group (median 13.0 ng/mL, IQR [11.5–14.5]). The CSF BSG concentration was lower in the CAA population (*p* = 0.036) and after exclusion of the outlier, the difference between the populations became more significant (CAA group: median 11.0 ng/mL, IQR [10.1–13.8]; *p* = 0.018, Figure 2A). The outlier was excluded in further analysis. CSF BSG levels remained significantly decreased in CAA after correction for age and sex (*p* = 0.03).

We observed no difference in CSF BSG levels between CAA patients with or without prior ICH (10.5 ng/mL, IQR [9.8–13.7]) versus 11.4 ng/mL (IQR [10.2–14.0]) respectively, *p* = 0.366 (Figure 2A). We found no correlation between the Qalb and CSF BSG levels in both the control (*ρ*_sp_ = −0.21, *p* = 0.32) and CAA participants (*ρ*_sp_ = 0.23, *p* = 0.20; Figure 2C), and no correlation between BSG and age (control *ρ*_sp_ = 0.20, *p* = 0.31; CAA group *ρ*_sp_ = −0.08, *p* = 0.64; Figure 2D, Table 3).

**TABLE 3:** Associations of CSF BSG with different independent variables in controls and patients with CAA.

	Controls (*n* = 27)		CAA (*n* = 39)	
	Spearman coefficient (*ρ*_sp_)	*p*	Spearman coefficient (*ρ*_sp_)	*p*
Age	0.20	0.31	−0.08	0.64
Qalb	−0.21	0.32	0.24	0.20
CSF Aβ40	0.80	**< 0.001**	0.55	**< 0.001**
CSF Aβ42	0.22	0.30	0.21	0.22
CSF t‐tau	0.87	**0.001**	0.64	**< 0.001**
CSF p‐tau	0.84	**0.001**	0.67	**< 0.001**
CAA‐related SVD burden score	0.356	0.81	0.035	0.841
Combined control and CAA subcohort; *n* = 16; controls *n* = 5; CAA patients; *n* = 11.				

	Spearman coefficient (*ρ*_sp_)		*p*	
CSF MMP2	−0.09		0.75	
CSF MMP9	0.38		0.14	
CSF MMP14	0.92		**< 0.001**	

*Note:* Bold values represent significant *p*‐values.

Abbreviations: Aβ, amyloid‐beta; CAA, cerebral amyloid angiopathy; CMB, cerebral microbleeds; Qalb, CSF/serum albumin quotient; MMP, matrix metalloproteinase; p‐tau, phosphorylated tau; *ρ*_sp_, Spearman's rank correlation coefficient; SVD, small vessel disease; t‐tau, total tau.

The CSF BSG concentration did not correlate with the MRI‐based CAA‐related SVD burden score in the CAA group (*ρ*_sp_ = 0.035; *p* = 0.841; control *ρ*_sp_ = 0.356, *p* = 0.81; Table 3). Also, the median BSG levels did not differ between the CAA patients without CMBs (*n* = 2, median 14.13 ng/mL, IQR [12.72–14.13], CAA patients with one to nine CMBs (*n* = 20, 10.33 ng/mL, IQR [9.17–12.02] and more than nine CMBs (*n* = 17, 11.84 ng/mL, IQR [10.34–14.11], *p* = 0.78).

The CSF BSG levels strongly correlated with CSF Aβ40 levels (controls: *ρ*_sp_ = 0.80, *p* < 0.001; CAA: *ρ*_sp_ = 0.55, *p* < 0.001) (Figure 2E) but not with Aβ42 levels (controls: *ρ*_s*p*_ = 0.22, *p* = 0.30; CAA: *ρ*_sp_ = 0.21, *p* = 0.22) (Table 3). Furthermore, CSF BSG concentrations correlated with CSF t‐tau (controls: *ρ*_sp_ = 0.77, *p* < 0.001; CAA patients: *ρ*_sp_ = 0.64, *p* < 0.001) and p‐tau levels (controls: *ρ*_sp_ = 0.84, *p* < 0.001; CAA patients: *ρ*_sp_ = 0.67, *p* < 0.001) (Table 3).

In CAA cases with a negative ATN profile (CAA + ATN−), we observed a significantly lower median BSG concentration (10.3 ng/mL, IQR [9.2–12.4]) in comparison to CAA ATN‐positive cases (12.1 ng/mL, IQR [10.8–14.58], *p* = 0.037) and the control population (13.0 ng/mL, IQR [11.6–14.5], *p* = 0.001, Figure 2B). Age and sex were nonsignificant covariates in this comparison (respectively, *p* = 0.96 and *p* = 0.925).

CSF concentrations of MMP2, MMP9 and MMP14 were only available in a small subpopulation. Due to the small size of this cohort (*n* = 16), we exploratively calculated the correlation of BSG with the MMP concentrations. MMP14, but not MMP2 and MMP9, showed a significant correlation with the BSG CSF levels (*ρ*_sp_ = 0.92, *p* < 0.001); see Figure 2F and Table 3.

## 4 Discussion

In our study combining both histopathological and in vivo assessment of BSG in patients with CAA and matched controls, we could demonstrate: (1) increased BSG expression in the cortical vasculature of CAA patients compared with the control; (2) increased BSG expression in CAA‐NH cases in comparison to both CAA‐ICH and control cases; (3) decreased BSG CSF concentration in patients with sCAA, both with and without prior ICH, compared with controls; and (4) BSG expression by choroid plexus epithelium of the BCSFB.

Our observation of strictly cortical vascular BSG expression has been reported before [37, 39, 49], although other reports of nonvascular BSG in cortical tissue have been described as well [50, 51]. In ad and control brains, BSG immunoreactivity has been described in neuronal axons and capillaries [50]. In mice brains, BSG was upregulated in microglia and astrocytes, in addition to the endothelial cells [50]. Our results that CAA cases have more abundant BSG expression in the cortical vasculature compared with the healthy control group are in line with previous literature regarding ad cases, where increased cortical vascular BSG immunoreactivity was reported [51]. No mention of CAA co‐pathology was made in this study, but it is likely that the majority of the ad cases in this cohort had some CAA‐positive vessels, based on the high prevalence of CAA in ad cases [52]. Interestingly, it has been described that cortical vessels from patients with mild cognitive impairment and Alzheimer's disease (ad) had less BSG in Aβ‐positive vessels compared with Aβ‐negative vessels [53]. These findings emphasise that CAA could affect BSG expression levels and that this could differ between the cortical capillaries of CAA patients and CAA‐affected penetrating vessels of larger size.

Remarkably, cortical BSG expression was higher in cases with CAA‐NH compared with controls. BSG expression may be upregulated in response to Aβ42‐induced production of reactive oxygen species [54]. Consequently, BSG may induce expression of Aβ‐degrading MMPs, reducing the amyloid‐induced stress on the vasculature and delaying the occurrence of a haemorrhage, potentially providing an explanation for the observed increased cerebral BSG expression in the CAA cases without a haemorrhage. In contrast, we found normal expression levels of BSG in CAA‐ICH patients. This could be due to an inverse interaction between MMPs and BSG; that is, BSG can be cleaved by upregulated MMP14 after the occurrence of an ICH [55, 56], which may explain why BSG expression in CAA‐ICH is lower than in CAA‐NH.

In both our IHC and fluid‐biomarker results, we observed neither a correlation between the %areaBSG and the Olichney CAA severity score, nor a correlation between the CSF BSG levels and the number of CMB or CAA‐related SVD burden score. This seems to indicate that BSG expression is linked to the presence of vascular Aβ rather than the severity of vascular Aβ depositions, presence of CapCAA and MRI‐based vascular dysfunction biomarkers. Because this study is, to our knowledge, the first to describe a potential role of BSG in CAA pathology and CAA‐related haemorrhage with immunohistochemistry and CSF concentrations, no other studies were available to compare, and follow‐up studies will aid in establishing these observations and are required prior to a mechanistic conclusion.

Similar to the decreased levels of Aβ40 in the CSF and increased presence of Aβ40 within the cerebral vasculature that are observed in CAA, the reduced CSF BSG levels in the CAA cohort (CAA‐NH and CAA‐ICH combined) may be linked to the observed increased vascular localisation of BSG. The reduced CSF BSG levels could be explained by reduced cleavage from the endothelial cell membrane. Cleavage may be mediated by MMP14, and indeed, we found a strong correlation between concentrations of BSG and MMP14 in CSF, which has been described previously as well [57]. So, this correlation between BSG and MMP14 could be a partial self‐enhancing mechanism. However, this explorative part of this study regarding the relation between MMPs and BSG in the context of CAA requires mechanistic research studies.

In both the CAA and control population, we found a strong positive correlation between CSF BSG and Aβ40 levels, but not with the amyloid plaque‐associated Aβ42 levels. We can only speculate what causes this correlation. While it has been described that Aβ42 can induce BSG expression, it is currently unknown whether Aβ40 can do so as well [54]. If this mechanism applies, it will connect the observed increased expression with the well‐known deposition of Aβ40 in CAA [2]. Future in vitro experiments may unravel mechanistic relations between BSG and Aβ40. The correlation between CSF BSG with both total‐ and phosphorylated‐tau is in line with the decreased BSG levels in the CAA + ATN− patients compared with the CAA + ATN+ patients. This may suggest that the decrease in CSF BSG levels in CAA is partially counteracted by a still to be explored mechanism of ad co‐pathology.

In addition to vascular BSG immunoreactivity, we also observed BSG expression by choroid plexus epithelium. This indicates that not only the endothelial cells of the BBB but also cells of the BCSFB may contribute to CSF concentrations. However, no abnormalities of the BCSFB are currently described in CAA pathology because it has been previously described that the volume of the choroid plexus is not increased in CAA patients compared with control patients [58]. Furthermore, in this study, the albumin ratio was not increased in CAA, as described before [46]. Therefore, we conclude that the aberrant CSF BSG levels are primarily affected by alterations at the BBB and are only minimally affected by barriers other than the BBB, such as the BCSFB. Moreover, a significant influence of peripheral BSG from the blood circulation is unlikely because the BSG concentration in blood is approximately twofold lower than in CSF [38]. Therefore, we propose that the altered BSG concentration in CSF in CAA reflects levels in the cortical vasculature and may be a proxy for the BBB function.

A limitation of this study is the limited number of cases included in the immunohistochemistry study. To increase the group sizes, we collected tissues from multiple sources. Although we could not demonstrate confounding effects, we cannot rule out that tissues have been treated differently between centres, especially with different fixation protocols. Furthermore, our IHC‐cohort was limited to control and CAA cases and did not include other SVD diseases to rule out possible mechanisms related to general SVD. Moreover, information about *APOE ɛ4* carriership and the presence of CapCAA was not available for all cases. However, we were able to perform an explorative analysis on these characteristics. BSG staining seems to be independent of *APOE ɛ4* and the presence of CapCAA. Strengths of our study include the unique cohorts of CSF collected from clinically well‐defined patients with CAA and controls, and of brain tissue from CAA patients either with or without concomitant ICH.

The CSF concentrations of BSG may be related to altered expression in the cerebral vasculature; the BSG concentrations in CSF may thus (indirectly) reflect BBB changes in CAA, although a contribution of choroid plexus epithelial BSG cannot be entirely excluded. Independent cohorts are needed to replicate these observations before we can conclude that reduced CSF concentrations of BSG may indicate an alteration of the BBB in patients with CAA.

## Author Contributions

A.S., H.B.K. and M.M.V. contributed to the conception and design of the study; A.M.D.K., F.H.B.M.S. and C.J.M.K. were responsible for the recruitment of patients and the collection of patient data. L.J., B.K., H.B.K. and M.M.V. were involved in the collection of human brain tissue. A.S., D.B., L.J., A.M.D.K., H.B.K. and M.M.V. contributed to data acquisition. A.S. performed data analysis. A.S., D.B., H.K.B. and M.M.V. interpreted the data. A.S. wrote the first manuscript draft. All authors reviewed and contributed to the manuscript and approved the final version.

## Ethics Statement

Brain tissue analysis: Samples obtained from the NBB, Netherlands Institute for Neuroscience, Amsterdam (NBB; Ref. No. 2009/148, open access: www.brainbank.nl) had been collected from donors that had provided written informed consent for the use of autopsy material and clinical information for research purposes, the study was performed in accordance with local regulations and approved by the medical research ethics committee of the UMCU (Ref. No. 17‐092). The use of autopsy materials from the Radboudumc was approved by the local ethics committee (Ref. No. 2015‐2215). Samples from the IBB Neurobiobank of the Institute Born‐Bunge (IBB) with FAMHP registration ID BBI90113, which is subject to biannual evaluation by the local Ethics Committee of University Hospital Antwerp (UZA/University of Antwerp (UAntwerp), Belgium, approved reference 19/13/166. Body fluid analysis: Lumbar‐ and venipunctures were performed after informed consent in the context of studies on biomarkers for CAA, which were approved by the local medical ethics committee of the RUMC (Ref. No. 2017‐3810, 2017‐3650). Samples were used anonymously in accordance with the Code of Conduct of the Federation of Medical Scientific Societies in the Netherlands and the 1964 Declaration of Helsinki.

## Conflicts of Interest

The authors declare no conflicts of interest.

## Funding

This work was supported by ZonMW (10510032120006, 733050822, 10510032120003), National Institutes of Health (5R01NS104147‐02), Stichting Alkemade‐Keuls, Maag‐Lever‐Darm‐stichting (WOO 2105), Parkinson NL (P2‐21‐18), and Alzheimer Nederland (WE.03‐2022‐17).

## Data Availability

The data that support the findings of this study are available from the corresponding author upon reasonable request.

## References

[1] A. Charidimou , G. Boulouis , M. E. Gurol , et al., “Emerging Concepts in Sporadic Cerebral Amyloid Angiopathy,” Brain 140 (2017): 1829–1850. 28334869 10.1093/brain/awx047 PMC6059159

[2] S. M. Greenberg , B. J. Bacskai , M. Hernandez‐Guillamon , J. Pruzin , R. Sperling , and S. J. van Veluw , “Cerebral Amyloid Angiopathy and Alzheimer Disease—One Peptide, Two Pathways,” Nature Reviews. Neurology 16 (2020): 30–42. 31827267 10.1038/s41582-019-0281-2 PMC7268202

[3] M. J. H. Wermer and S. M. Greenberg , “The Growing Clinical Spectrum of Cerebral Amyloid Angiopathy,” Current Opinion in Neurology 31 (2018): 28–35. 29120920 10.1097/WCO.0000000000000510

[4] M. G. Kozberg , I. Yi , W. M. Freeze , et al., “Blood‐Brain Barrier Leakage and Perivascular Inflammation in Cerebral Amyloid Angiopathy,” Brain Communications 4 (2022): fcac245. 36267331 10.1093/braincomms/fcac245 PMC9576155

[5] W. M. Freeze , B. J. Bacskai , M. P. Frosch , et al., “Blood‐Brain Barrier Leakage and Microvascular Lesions in Cerebral Amyloid Angiopathy,” Stroke 50 (2019): 328–335. 30661497 10.1161/STROKEAHA.118.023788 PMC6415745

[6] A. Charidimou , G. Boulouis , M. P. Frosch , et al., “The Boston Criteria Version 2.0 for Cerebral Amyloid Angiopathy: A Multicentre, Retrospective, MRI‐Neuropathology Diagnostic Accuracy Study,” Lancet Neurology 21 (2022): 714–725. 35841910 10.1016/S1474-4422(22)00208-3 PMC9389452

[7] L. Jakel , K. Claassen , A. M. De Kort , et al., “Decreased Microvascular Claudin‐5 Levels in Cerebral Amyloid Angiopathy Associated With Intracerebral Haemorrhage,” Brain Pathology 34 (2024): e13270. 38763889 10.1111/bpa.13270 PMC11483184

[8] A. V. Andjelkovic , M. Situ , A. F. Citalan‐Madrid , S. M. Stamatovic , J. Xiang , and R. F. Keep , “Blood‐Brain Barrier Dysfunction in Normal Aging and Neurodegeneration: Mechanisms, Impact, and Treatments,” Stroke 54 (2023): 661–672. 36848419 10.1161/STROKEAHA.122.040578 PMC9993074

[9] H. Kadry , B. Noorani , and L. Cucullo , “A Blood‐Brain Barrier Overview on Structure, Function, Impairment, and Biomarkers of Integrity,” Fluids and Barriers of the CNS 17 (2020): 69. 33208141 10.1186/s12987-020-00230-3 PMC7672931

[10] A. P. Viggars , S. B. Wharton , J. E. Simpson , et al., “Alterations in the Blood Brain Barrier in Ageing Cerebral Cortex in Relationship to Alzheimer‐Type Pathology: A Study in the MRC‐CFAS Population Neuropathology Cohort,” Neuroscience Letters 505 (2011): 25–30. 21970975 10.1016/j.neulet.2011.09.049

[11] H. Tayler , J. S. Miners , O. Guzel , R. MacLachlan , and S. Love , “Mediators of Cerebral Hypoperfusion and Blood‐Brain Barrier Leakiness in Alzheimer's Disease, Vascular Dementia and Mixed Dementia,” Brain Pathology 31 (2021): e12935. 33410232 10.1111/bpa.12935 PMC8412075

[12] M. J. Thrippleton , W. H. Backes , S. Sourbron , et al., “Quantifying Blood‐Brain Barrier Leakage in Small Vessel Disease: Review and Consensus Recommendations,” Alzheimers Dement 15 (2019): 840–858. 31031101 10.1016/j.jalz.2019.01.013 PMC6565805

[13] S. Chalbot , H. Zetterberg , K. Blennow , et al., “Blood‐Cerebrospinal Fluid Barrier Permeability in Alzheimer's Disease,” Journal of Alzheimer's Disease 25 (2011): 505–515. 10.3233/JAD-2011-101959 PMC3139450 21471645

[14] E. Giacopuzzi Grigoli , F. Solca , I. Milone , et al., “Cerebrospinal Fluid/Serum Albumin Quotient (Q‐Alb) Is Not Increased in Alzheimer's Disease Compared to Neurological Disease Controls: A Retrospective Study on 276 Patients,” Neurological Sciences 44 (2023): 709–713. 36441343 10.1007/s10072-022-06530-w

[15] H. Reiber , “Cerebrospinal Fluid—Physiology, Analysis and Interpretation of Protein Patterns for Diagnosis of Neurological Diseases,” Multiple Sclerosis 4 (1998): 99–107. 9762655 10.1177/135245859800400302

[16] M. Andersson , J. Alvarez‐Cermeño , G. Bernardi , et al., “Cerebrospinal Fluid in the Diagnosis of Multiple Sclerosis: A Consensus Report,” Journal of Neurology, Neurosurgery, and Psychiatry 57 (1994): 897–902. 8057110 10.1136/jnnp.57.8.897 PMC1073070

[17] H. Reiber and K. Felgenhauer , “Protein Transfer at the Blood Cerebrospinal Fluid Barrier and the Quantitation of the Humoral Immune Response Within the Central Nervous System,” Clinica Chimica Acta 163 (1987): 319–328. 10.1016/0009-8981(87)90250-6 3581475

[18] Z. Redzic , “Molecular Biology of the Blood‐Brain and the Blood‐Cerebrospinal Fluid Barriers: Similarities and Differences,” Fluids and Barriers of the CNS 8 (2011): 3. 21349151 10.1186/2045-8118-8-3 PMC3045361

[19] G. Carstens , M. M. Verbeek , U. K. Rohlwink , A. A. Figaji , L. Te Brake , and A. van Laarhoven , “Metabolite Transport Across Central Nervous System Barriers,” Journal of Cerebral Blood Flow and Metabolism 44 (2024): 271678X241241908. 10.1177/0271678X241241908 PMC11179608 38546534

[20] I. Kratzer , A. Vasiljevic , C. Rey , et al., “Complexity and Developmental Changes in the Expression Pattern of Claudins at the Blood‐CSF Barrier,” Histochemistry and Cell Biology 138 (2012): 861–879. 22886143 10.1007/s00418-012-1001-9 PMC3483103

[21] A. A. Bergen , S. Kaing , J. B. ten Brink , B. Netherlands Brain , T. G. Gorgels , and S. F. Janssen , “Gene Expression and Functional Annotation of Human Choroid Plexus Epithelium Failure in Alzheimer's Disease,” BMC Genomics 16 (2015): 956. 26573292 10.1186/s12864-015-2159-z PMC4647590

[22] C. Avolio , F. Giuliani , G. M. Liuzzi , et al., “Adhesion Molecules and Matrix Metalloproteinases in Multiple Sclerosis: Effects Induced by Interferon‐Beta,” Brain Research Bulletin 61 (2003): 357–364. 12909305 10.1016/s0361-9230(03)00098-4

[23] S. Horstmann , L. Budig , H. Gardner , et al., “Matrix Metalloproteinases in Peripheral Blood and Cerebrospinal Fluid in Patients With Alzheimer's Disease,” International Psychogeriatrics 22 (2010): 966–972. 20561382 10.1017/S1041610210000827

[24] S. E. Lakhan , A. Kirchgessner , D. Tepper , and A. Leonard , “Matrix Metalloproteinases and Blood‐Brain Barrier Disruption in Acute Ischemic Stroke,” Frontiers in Neurology 4 (2013): 32. 23565108 10.3389/fneur.2013.00032 PMC3615191

[25] D. K. Kaushik , J. N. Hahn , and V. W. Yong , “EMMPRIN, an Upstream Regulator of MMPs, in CNS Biology,” Matrix Biology 44‐46 (2015): 138–146. 10.1016/j.matbio.2015.01.018 25644103

[26] Y. Yang , E. Y. Estrada , J. F. Thompson , W. Liu , and G. A. Rosenberg , “Matrix Metalloproteinase‐Mediated Disruption of Tight Junction Proteins in Cerebral Vessels Is Reversed by Synthetic Matrix Metalloproteinase Inhibitor in Focal Ischemia in Rat,” Journal of Cerebral Blood Flow & Metabolism 27 (2007): 697–709. 16850029 10.1038/sj.jcbfm.9600375

[27] T. Higashida , C. W. Kreipke , J. A. Rafols , et al., “The Role of Hypoxia‐Inducible Factor‐1α, Aquaporin‐4, and Matrix Metalloproteinase‐9 in Blood‐Brain Barrier Disruption and Brain Edema After Traumatic Brain Injury,” Journal of Neurosurgery 114 (2011): 92–101. 20617879 10.3171/2010.6.JNS10207

[28] Y. Yang and G. A. Rosenberg , “MMP‐Mediated Disruption of Claudin‐5 in the Blood–Brain Barrier of Rat Brain After Cerebral Ischemia,” in Claudins: Methods and Protocols, ed. K. Turksen (Humana Press, 2011), 333–345. 10.1007/978-1-61779-185-7_24 PMC4950933 21717368

[29] L. Jakel , A. M. De Kort , A. Stellingwerf , et al., “Altered Brain Expression and Cerebrospinal Fluid Levels of TIMP4 in Cerebral Amyloid Angiopathy,” Acta Neuropathologica Communications 12 (2024): 103. 38915119 10.1186/s40478-024-01823-x PMC11194996

[30] M. Vervuurt , A. M. de Kort , L. Jäkel , et al., “Decreased Ratios of Matrix Metalloproteinases to Tissue‐Type Inhibitors in Cerebrospinal Fluid in Sporadic and Hereditary Cerebral Amyloid Angiopathy,” Alzheimer's Research & Therapy 15 (2023): 26. 10.1186/s13195-023-01171-3 PMC9885599 36717932

[31] L. Jakel , H. B. Kuiperij , L. P. Gerding , et al., “Disturbed Balance in the Expression of MMP9 and TIMP3 in Cerebral Amyloid Angiopathy‐Related Intracerebral Haemorrhage,” Acta Neuropathologica Communications 8 (2020): 99. 32631441 10.1186/s40478-020-00972-z PMC7336459

[32] L. Zhao , M. Arbel‐Ornath , X. Wang , et al., “Matrix Metalloproteinase 9–Mediated Intracerebral Hemorrhage Induced by Cerebral Amyloid Angiopathy,” Neurobiology of Aging 36 (2015): 2963–2971. 26248866 10.1016/j.neurobiolaging.2015.07.016 PMC4609585

[33] K. S. Vetrivel , X. Zhang , X. Meckler , et al., “Evidence That CD147 Modulation of Beta‐Amyloid (Abeta) Levels Is Mediated by Extracellular Degradation of Secreted Abeta,” Journal of Biological Chemistry 283 (2008): 19489–19498. 18456655 10.1074/jbc.M801037200 PMC2443668

[34] F. Desmarais , V. Hervé , K. F. Bergeron , et al., “Cerebral Apolipoprotein D Exits the Brain and Accumulates in Peripheral Tissues,” International Journal of Molecular Sciences 22 (2021): 4118. 33923459 10.3390/ijms22084118 PMC8073497

[35] O. Najyb , L. Brissette , and E. Rassart , “Apolipoprotein D Internalization Is a Basigin‐Dependent Mechanism,” Journal of Biological Chemistry 290 (2015): 16077–16087. 25918162 10.1074/jbc.M115.644302 PMC4481210

[36] M. Hernandez‐Guillamon , S. Mawhirt , S. Blais , et al., “Sequential Amyloid‐Beta Degradation by the Matrix Metalloproteases MMP‐2 and MMP‐9,” Journal of Biological Chemistry 290 (2015): 15078–15091. 25897080 10.1074/jbc.M114.610931 PMC4463451

[37] T. Sameshima , K. Nabeshima , B. P. Toole , et al., “Expression of Emmprin (CD147), a Cell Surface Inducer of Matrix Metalloproteinases, in Normal Human Brain and Gliomas,” International Journal of Cancer 88 (2000): 21–27. 10962435 10.1002/1097-0215(20001001)88:1<21::aid-ijc4>3.0.co;2-s

[38] D. K. Kaushik , H. Y. Yong , J. N. Hahn , et al., “Evaluating Soluble EMMPRIN as a Marker of Disease Activity in Multiple Sclerosis: Studies of Serum and Cerebrospinal Fluid,” PLoS ONE 11 (2016): e0163802. 27727297 10.1371/journal.pone.0163802 PMC5058493

[39] A. Patrizz , S. J. Doran , A. Chauhan , et al., “EMMPRIN/CD147 Plays a Detrimental Role in Clinical and Experimental Ischemic Stroke,” Aging (Albany NY) 12 (2020): 5121–5139. 32191628 10.18632/aging.102935 PMC7138568

[40] G. S. Butler and C. M. Overall , “Updated Biological Roles for Matrix Metalloproteinases and New “Intracellular” Substrates Revealed by Degradomics,” Biochemistry 48 (2009): 10830–10845. 19817485 10.1021/bi901656f

[41] J. M. Olichney , L. A. Hansen , D. Galasko , et al., “The Apolipoprotein E Epsilon 4 Allele Is Associated With Increased Neuritic Plaques and Cerebral Amyloid Angiopathy in Alzheimer's Disease and Lewy Body Variant,” Neurology 47 (1996): 190–196. 8710076 10.1212/wnl.47.1.190

[42] J. Sauvola and M. Pietikäinen , “Adaptive Document Image Binarization,” Pattern Recognition 33 (2000): 225–236.

[43] J. Linn , A. Halpin , P. Demaerel , et al., “Prevalence of Superficial Siderosis in Patients With Cerebral Amyloid Angiopathy,” Neurology 74 (2010): 1346–1350. 20421578 10.1212/WNL.0b013e3181dad605 PMC2875936

[44] A. M. De Kort , K. Kaushik , H. B. Kuiperij , et al., “The Relation of a Cerebrospinal Fluid Profile Associated With Alzheimer's Disease With Cognitive Function and Neuropsychiatric Symptoms in Sporadic Cerebral Amyloid Angiopathy,” Alzheimer's Research & Therapy 16 (2024): 99. 10.1186/s13195-024-01454-3 PMC11069247 38704569

[45] A. M. De Kort , H. B. Kuiperij , T. M. Marques , et al., “Decreased Cerebrospinal Fluid Amyloid beta 38, 40, 42, and 43 Levels in Sporadic and Hereditary Cerebral Amyloid Angiopathy,” Annals of Neurology 93 (2023): 1173–1186. 36707720 10.1002/ana.26610 PMC10238617

[46] A. M. De Kort , H. B. Kuiperij , I. Kersten , et al., “Normal Cerebrospinal Fluid Concentrations of PDGFRbeta in Patients With Cerebral Amyloid Angiopathy and Alzheimer's Disease,” Alzheimer's & Dementia 18 (2022): 1788–1796. 10.1002/alz.12506 PMC9787758 34874603

[47] C. R. Jack, Jr. , D. A. Bennett , K. Blennow , et al., “NIA‐AA Research Framework: Toward a Biological Definition of Alzheimer's Disease,” Alzheimer's & Dementia 14 (2018): 535–562. 10.1016/j.jalz.2018.02.018 PMC5958625 29653606

[48] U. Andreasson , A. Perret‐Liaudet , L. J. van Waalwijk van Doorn , et al., “A Practical Guide to Immunoassay Method Validation,” Frontiers in Neurology 6 (2015): 179. 26347708 10.3389/fneur.2015.00179 PMC4541289

[49] T. Sameshima , K. Nabeshima , B. P. Toole , et al., “Correlation of Emmprin Expression in Vascular Endothelial Cells With Blood‐Brain‐Barrier Function: A Study Using Magnetic Resonance Imaging Enhanced by Gd‐DTPA and Immunohistochemistry in Brain Tumors,” Virchows Archiv 442 (2003): 577–584. 12719975 10.1007/s00428-003-0801-7

[50] Y. Liu , Q. Bai , V. W. Yong , and M. Xue , “EMMPRIN Promotes the Expression of MMP‐9 and Exacerbates Neurological Dysfunction in a Mouse Model of Intracerebral Hemorrhage,” Neurochemical Research 47 (2022): 2383–2395. 35608790 10.1007/s11064-022-03630-z

[51] J. Nahalkova , I. Volkmann , M. Aoki , et al., “CD147, a Gamma‐Secretase Associated Protein Is Upregulated in Alzheimer's Disease Brain and Its Cellular Trafficking Is Affected by Presenilin‐2,” Neurochemistry International 56 (2010): 67–76. 19751784 10.1016/j.neuint.2009.09.003

[52] L. Jakel , A. M. De Kort , C. J. M. Klijn , F. Schreuder , and M. M. Verbeek , “Prevalence of Cerebral Amyloid Angiopathy: A Systematic Review and Meta‐Analysis,” Alzheimers Dement 18 (2022): 10–28. 34057813 10.1002/alz.12366 PMC9290643

[53] D. Leitner , T. Kavanagh , E. Kanshin , et al., “Differences in the Cerebral Amyloid Angiopathy Proteome in Alzheimer's Disease and Mild Cognitive Impairment,” Acta Neuropathologica 148 (2024): 9. 39039355 10.1007/s00401-024-02767-1 PMC11263258

[54] L. J. Kanyenda , G. Verdile , R. Martins , et al., “Is Cholesterol and Amyloid‐Beta Stress Induced CD147 Expression a Protective Response? Evidence That Extracellular Cyclophilin A Mediated Neuroprotection Is Reliant on CD147,” Journal of Alzheimer's Disease 39 (2014): 545–556. 10.3233/JAD-131442 24217276

[55] N. Egawa , N. Koshikawa , T. Tomari , K. Nabeshima , T. Isobe , and M. Seiki , “Membrane Type 1 Matrix Metalloproteinase (MT1‐MMP/MMP‐14) Cleaves and Releases a 22‐kDa Extracellular Matrix Metalloproteinase Inducer (EMMPRIN) Fragment From Tumor Cells,” Journal of Biological Chemistry 281 (2006): 37576–37585. 17050542 10.1074/jbc.M606993200

[56] Y. Liu , L. Qi , Z. Li , V. W. Yong , and M. Xue , “Crosstalk Between Matrix Metalloproteinases and Their Inducer EMMPRIN/CD147: A Promising Therapeutic Target for Intracerebral Hemorrhage,” Translational Stroke Research 16 (2023): 557–567. 38100014 10.1007/s12975-023-01225-6

[57] T. Sameshima , K. Nabeshima , B. P. Toole , et al., “Glioma Cell Extracellular Matrix Metalloproteinase Inducer (EMMPRIN) (CD147) Stimulates Production of Membrane‐Type Matrix Metalloproteinases and Activated Gelatinase A in Co‐Cultures With Brain‐Derived Fibroblasts,” Cancer Letters 157 (2000): 177–184. 10936678 10.1016/s0304-3835(00)00485-7

[58] L. Zhao , L. Liu , M. Lin , et al., “Relationship Between Cerebrospinal Fluid Circulation Markers, Brain Degeneration, and Cognitive Impairment in Cerebral Amyloid Angiopathy,” Frontiers in Aging Neuroscience 17 (2025): 1549072. 40330595 10.3389/fnagi.2025.1549072 PMC12053238

